# Special issue on hypoxia

**DOI:** 10.1038/s12276-019-0257-8

**Published:** 2019-06-20

**Authors:** Eui-Ju Yeo

**Affiliations:** Department of Biochemistry, College of Medicine, Gachon University, Incheon, 21999 South Korea

The critical roles of oxygen (O_2_) in aerobic respiration and metabolism are indisputable. Oxygen acts as the final electron acceptor in the mitochondrial electron transport chain to generate ATP within eukaryotic cells^1^. Therefore, an adequate concentration of oxygen is required by eukaryotic cells to maintain a variety of biological activities and ensure survival. When oxygen levels in the whole body or local tissues are severely reduced, hypoxia occurs, leading to a metabolic crisis and threatening physiological functions and viability. Because of the importance of oxygen, eukaryotes have developed an efficient and rapid oxygen-sensing system: hypoxia-inducible factors (HIFs)^2^. Hypoxic responses are controlled by HIF stabilization, which induces the expression of more than 100 downstream target genes to increase the oxygen supply and support anaerobic ATP generation in eukaryotic cells. Over the past two decades, HIF isotypes and their functions, and binding factors/coactivators, and the regulatory mechanisms by which cells sense hypoxia and transduce a signal to the HIF pathway have been intensively studied by many researchers and expanded our knowledge of hypoxia to the cellular and molecular levels. The HIF pathway is summarized in the introduction section of the review by Holger K. Eltzschig’s group (The University of Texas Health Science Center) and in the first section (“Hypoxia and the HIF pathway”) of the review by Eui-Ju Yeo (Gachon University).

In humans, oxygen is exchanged in the alveoli of the lungs. Over 95% of the oxygen delivered into the capillary vessels binds to hemoglobin. The heart pumps blood containing oxygenated hemoglobin, which is crucial for the biological function of organs and cells, to the periphery. Any failure during this process can cause hypoxia in organs and cells. Interestingly, hypoxia and tissue inflammation are closely related to each other in various organ injuries^3,4^. Hypoxia can activate the nuclear factor κB (NF-κB) pathway, an HIF-independent signaling pathway. IκBα is phosphorylated during hypoxia, resulting in the degradation of IκBα and the activation of NF-κB^5^. In fact, many studies demonstrate that while hypoxia causes tissue inflammation, HIF stabilization can reduce tissue inflammation and promote its repair^6–8^. HIF may elicit the upregulation of transcriptional cascades important for tissue protection and adaptation. The HIF-driven adenosine signaling pathway is well-known to serve as a protective mechanism and provide ischemic tolerance in tissues exposed to acute hypoxia^6,9^. Upon hypoxic cellular and tissue injury, stabilized HIF1A binds to the promoter region of ecto-5′-nucleotidase (CD73) and increases the CD73 enzyme levels, resulting in increases in the levels of extracellular adenosine and ATP/ADP^10^. Extracellular adenosine can act directly as a signaling molecule working through adenosine receptors. Adenosine receptors 2B and 2A are direct targets of HIF1A and HIF2A, respectively^11^. Indeed, increasing extracellular adenosine levels by the inhibition of equilibrative nucleoside transporters results in protection from inflammation^12^.

Due to the undisputed biological importance and protective function of HIF and its downstream targets, hypoxia and the HIF signaling pathways are emerging as novel therapeutic options to treat various organ injuries. The review by Holger K. Eltzschig’s group highlights the current understanding of hypoxia signaling in different human diseases related to four different organ systems: the heart, lung, liver, and kidney. This review also discusses the divergent roles of HIFs in acute and chronic disease conditions in these four organ systems. In general, HIF stabilization by preconditioning/postconditioning or pharmacologic intervention confers a protective phenotype across all organs during acute conditions, as shown in various in vivo studies and human clinical trials. However, modulating the HIF pathway in chronic disease conditions seems to be more complex than in acute conditions, because the effects of HIF stabilization are controversial in different studies. Nonetheless, targeting the HIF signaling pathway in chronic disease conditions still holds promise in effectively managing or delaying the progression of disease. Finally, the review introduces some efforts to translate current knowledge about hypoxia signaling to clinical medicine. As our understanding of the pathophysiology of diseases and its relation to hypoxia signaling deepens, it will be possible to discover additional therapeutic targets and niches for intervention.

Hypoxia also contributes to functional decline during the aging process. The putative molecular mechanisms underlying the effects of hypoxia and HIF-1α on aging are discussed in the section “HIF-1α and aging” of the review by Eui-Ju Yeo. This section includes a discussion of cross-talk between HIF pathways and aging-associated signaling proteins, such as sirtuins, AMP-activated protein kinase, mechanistic target of rapamycin complex 1, UNC-51-like kinase 1, and NF-κB, in aging and aging-related diseases^13–16^.

In recent years, the effects of prenatal hypoxia and obstructive sleep apnea (OSA) have garnered interest due to their effect on accelerating the progression and increasing the severity of many diseases. Prenatal hypoxia leads to insufficient oxygen supply to the fetus during critical periods of brain development, which is one of the most important factors manifesting in early aging, mental retardation, and cognitive deficits at various postnatal stages^17^. An OSA, characterized by repeated episodes of complete or partial obstruction of the upper airway, causes cyclical hypoxemia-reoxygenation and stimulates chemoreceptors, resulting in chronic intermittent cellular hypoxia^18^. Long-term consequences of OSA contribute to many diseases, including cardiovascular diseases, metabolic diseases, neurological disorders, cancer, and aging^19^. Therefore, the pathophysiological consequences and clinical manifestations of prenatal hypoxia and OSA-induced chronic intermittent hypoxia are included in the review by Eui-Ju Yeo. It is likely that additional strategies targeting hypoxia-related signaling pathways will be helpful for the prevention of aging and aging-related diseases.

The basic unit of heterochromatin structure, termed the nucleosome, is composed of a histone octamer (two copies each of H2A, H2B, H3, and H4) wrapped by DNA. Posttranslational modifications of histones determine the 3-D structure of chromatin, affecting DNA replication and repair, splicing, and transcription. Posttranslational modifications of histones are determined by the balance between “writers” and “erasers”. For example, members of the histone H3 lysine 4 (H3K4) methyltransferase family, such as MLL3/KMT2C or MLL4/KMT2D, are examples of writers, which methylate H3K4 using S-adenosyl-methionine as the methyl donor. Jumonji domain-containing histone lysine demethylases (KDMs, e.g., JARID1C/KDM5C and UTX/KDM6A) are examples of erasers, which demethylate H3K4me3 or H3K4me2. KDMs use O_2_, α-ketoglutarate (α-KG), vitamin C, and Fe(II) as cosubstrates and cofactors. Therefore, metabolic control of histone modification depends on the availability of cosubstrates, coenzymes/cofactors, and inhibitors of the writers and erasers.

Interestingly, several studies showed that O_2_- and α-KG-dependent KDMs mediate signals from hypoxic tumor microenvironments and the metabolic status to nuclear chromatin. Hypoxia contributes to the inhibition of KDMs via multiple processes: (i) by limiting their substrate, O_2_ and (ii) by metabolic reprogramming to deplete their cosubstrate, α-KG, and increase the level of endogenous l-2-hydroxyglutarate (l-2HG), a competitive inhibitor of α-KG-dependent KDMs^20,21^. Under hypoxic conditions, l-2HG production is increased via catalytic activities of lactate dehydrogenase A and malate dehydrogenases 1 and 2, which are induced in an HIF-1α-dependent manner^20,22^. l-2HG is oxidized to α-KG by l-2HG dehydrogenase in mitochondria^23^. Therefore, defects in 2-HG dehydrogenases also induce l-2HG accumulation, which is associated with brain tumors^24^ and neurodegeneration^25^. Furthermore, the increased glutamine catabolism in tumors depletes the local glutamine supply, leading to a reduction in cytoplasmic glutamate and α-KG^26^. Therefore, glutamine depletion may increase the abundance of methylated histones via a reduction in the level of α-KG, a substrate of KDMs, and contributes to drug resistance and tumor heterogeneity^27^. The review by Hyunsung Park’s group discusses in detail recent advances regarding metabolic reprogramming of the α-KG balance by hypoxia.

However, genetic mutations in isocitrate dehydrogenases (*IDH1 and IDH2)* and D-3-phosphoglycerate dehydrogenase (*PHGDH*) result in a high level of D/R-2HG (termed oncometabolite), an enantiomer of l-2HG with similar inhibitory effects on O_2_- and α-KG-dependent KDMs, thus increasing the total abundance of methylated histones in various cancer cells. The Hyunsung Park’s group reviews recent advances regarding metabolic reprogramming of the α-KG balance by *IDH* mutation in cancers. Wild-type IDH1 and 2 are well-known to catalyze the oxidative decarboxylation of isocitrate to generate α-KG and CO_2_. Missense mutations in IDH1 and 2 result in new activities that further convert α-KG to D/R-2HG in various cancer cells^28^. A PHGDH also catalyzes the reduction of α-KG to D-2HG using NADH^29^. A PHGDH is frequently amplified, and 2-HG thus accumulates in breast cancer^30,31^. However, the mechanism through which histone methylation is related to cellular heterogeneity, resistance to chemotherapy and radiotherapy, and cancer progression remains poorly understood. In summary, additional strategies targeting the HIF pathway, hypoxia-related signaling pathways, and metabolic reprogramming will be helpful for the prevention and treatment of various clinical problems and aging-related diseases, including cancer.
